# Three-Dimensional Preoperative Planning in Reverse Shoulder Arthroplasty: A Systematic Review of Implant Positioning Accuracy and Clinical Outcomes

**DOI:** 10.7759/cureus.112080

**Published:** 2026-07-05

**Authors:** Konstantinos Zampetakis, Georgios Sakellaridis, Panagiotis Lepetsos, Anastasios Tsiotsias, Andreas Leonidou

**Affiliations:** 1 Department of Orthopaedics and Traumatology, The General Hospital of Heraklion "Venizeleio-Pananio", Heraklion, GRC; 2 School of Medicine, University of Crete, Heraklion, GRC; 3 School of Medicine, European University Cyprus, Nicosia, CYP; 4 Department of Orthopaedics and Traumatology, Athens Medical Center, Athens, GRC

**Keywords:** augmented reality, navigation, patient-specific instrumentation, preoperative planning, reverse shoulder arthroplasty, three-dimensional

## Abstract

Accurate implant positioning in reverse shoulder arthroplasty (RSA) is crucial for long-term survival and favourable postoperative outcomes. Three-dimensional (3D) preoperative planning, including patient-specific instrumentation (PSI) and navigation systems, has emerged in the past decade to increase implantation accuracy and efficacy. This review aimed to assess the radiological and clinical impact of 3D preoperative planning in RSA. The PubMed and Scopus databases (2010-2026) were systematically reviewed according to the PRISMA (Preferred Reporting Items for Systematic Reviews and Meta-Analyses) guidelines. Inclusion criteria involved English-language studies assessing 3D preoperative planning for RSA, with or without PSI or navigation, and reporting radiological and/or clinical outcomes. Data on study characteristics, patient demographics, RSA indications, 3D planning technology used, radiological outcomes, and functional outcomes were extracted and analyzed. A total of 635 RSAs across 11 studies were included, with a mean follow-up of 42.6 months. The most common indication for RSA was rotator cuff arthropathy. Seven of 11 studies used PSI, two of 11 studies used intraoperative navigation, and two of 11 studies assessed isolated 3D preoperative planning. Most comparative and non-comparative studies indicated significant improvement or high concordance with the preoperative plan in glenoid version, inclination, and entry point accuracy when 3D preoperative planning was used, especially when combined with PSI or navigation. 3D planning improved the accuracy of screw placement and length without increasing complications. Three studies assessed functional outcomes, where the differences between PSI-assisted and conventional techniques were inconsistent. No significant differences were detected in operating time and complication rates with the use of 3D preoperative planning. 3D preoperative planning techniques improve the accuracy of glenoid component and peripheral screw implantation in RSA. Evidence for superior clinical outcomes or differences in surgical parameters and complications remains inconsistent. Future high-quality research is needed to determine the clinical value of 3D planning modalities.

## Introduction and background

Reverse shoulder arthroplasty (RSA) has become one of the most successful surgical procedures addressing numerous complex shoulder pathologies [1]. While RSA was initially developed as a treatment option for chronic rotator cuff arthropathy in the elderly, its indications have substantially expanded and currently include primary glenohumeral arthritis, irreparable rotator cuff tears, pseudoparalysis, proximal humerus fractures, non-union or mal-union, and revision for previous arthroplasty [2,3]. The increasing use of RSA, especially among younger patients, has led to particular interest in factors influencing long-term implant survival and postoperative outcomes [4].

The placement of the glenoid component is considered one of the most challenging stages of the procedure. The presence of increased retroversion, pathological inclination, bone defects, or severe glenoid deformity may make it difficult to accurately position the glenoid base and negatively affect the stability and long-term survival of the implant [5]. Suboptimal placement has been associated with an increased risk of loosening, instability, scapular notching, reduced bone support, and early failure of the arthroplasty [6,7]. Despite the extensive use of computed tomography (CT) for preoperative planning, conventional two-dimensional (2D) assessment methods may not sufficiently capture the complexity of the glenoid anatomy, especially in cases of significant deformity [8].

Over the past decade, three-dimensional (3D) preoperative planning technologies have been developed to improve surgical accuracy in orthopaedic surgery [9]. Using 3D reconstructions from CT scans, the surgeon can more accurately evaluate the morphology of the native glenoid, plan the position and orientation of the glenoid component, select the appropriate implant type and size, and simulate different surgical scenarios in vitro [10]. In addition, patient-specific instrumentation (PSI), navigation and augmented reality systems have also been developed to more precisely reproduce preoperative planning during surgery, using patient-tailored surgical guides, real-time intraoperative feedback, and virtual overlays of the surgical field, respectively [11]. Although these technologies are increasingly used, the available data remain heterogeneous. Published studies have evaluated different technologies, different radiological and clinical outcomes, and a variety of surgical parameters and complications [10,12]. Furthermore, to date, it is unclear whether improved radiological accuracy translates into a significant functional benefit for patients or a reduction in complications and revisions.

The aim of this systematic review was to comprehensively evaluate the available 3D preoperative planning technologies in RSA, with or without PSI and/or navigation systems. The primary objective was to investigate their radiological effect on the accuracy of the glenoid component, entry point, and peripheral screw placement, while secondary objectives were to assess whether this effect translated into improved functional outcomes, surgical parameters, complications, and reoperations.

## Review

Methods

PubMed and Scopus databases were systematically searched from January 2010 to May 2026. The following search strategy was applied in both databases: ("reverse shoulder arthroplasty" OR "reverse total shoulder arthroplasty" OR RTSA OR RSA) AND (planning OR navigation OR "patient-specific instrumentation" OR PSI OR "three-dimensional" OR 3D). This systematic review was registered in PROSPERO (CRD420261393493) and was prepared in accordance with Preferred Reporting Items for Systematic Review and Meta-Analyses (PRISMA) 2020 guidelines [13]. Moreover, the PICOS (population, intervention, comparison, outcome, study type) question format was used to plan the analysis [14]. Duplicate records were removed using the Mendeley Reference Manager software (Elsevier, Amsterdam, Netherlands). The quality of the studies was assessed by two authors using the Cochrane RoB 2 (Risk of Bias 2) and the Newcastle-Ottawa Scale (NOS) [15,16]. In case of disparity between the two authors during the screening and data extraction process, a third reviewer was consulted to achieve consensus.

Inclusion Criteria

The population of interest was adults undergoing planned RSA using 3D preoperative planning software. This systematic review included studies evaluating 3D preoperative planning for RSA, with or without PSI or intraoperative navigation, and studies reporting clinical, radiological, surgical, or complication-related outcomes after RSA. Only studies in the English language, where full text was available, were included.

Exclusion Criteria

The exclusion criteria were studies not involving RSA, studies not using 3D preoperative planning and studies without extractable clinical or radiological outcomes. Studies with mixed shoulder arthroplasty populations (e.g., TSA and RSA) in which RSA-specific data could not be extracted were also excluded. This systematic review also excluded cadaveric, in vitro, biomechanical, or simulation studies as well as case reports, reviews, editorials, and conference abstracts without full data.

Study characteristics (study design, sample size, and country), patient demographics, indications for RSA, and the 3D planning techniques used, including the use of PSI and/or navigation, were documented. Moreover, control groups, where applicable, were documented, along with the evaluated outcomes, in each study. The measured outcomes were categorized into radiological outcomes (such as version error, inclination error, and entry point deviation), functional outcomes and complications.

Data Analysis

Due to significant heterogenicity across the included studies regarding study design, patient populations, 3D preoperative templating techniques and reported outcomes, no quantitative synthesis (meta-analysis) was performed. Instead, a structured narrative synthesis of the data was applied. Outcomes were grouped into radiological, functional, surgical, and complication-related categories. Within each category, findings were compared descriptively across studies, with emphasis on the consistency and direction of the effect of the interventions. Where possible, results were summarized with descriptive statistics and reporting the number of studies that demonstrated improvement, no difference, or worse outcomes.

Results

Search Strategy

**Figure 1:**
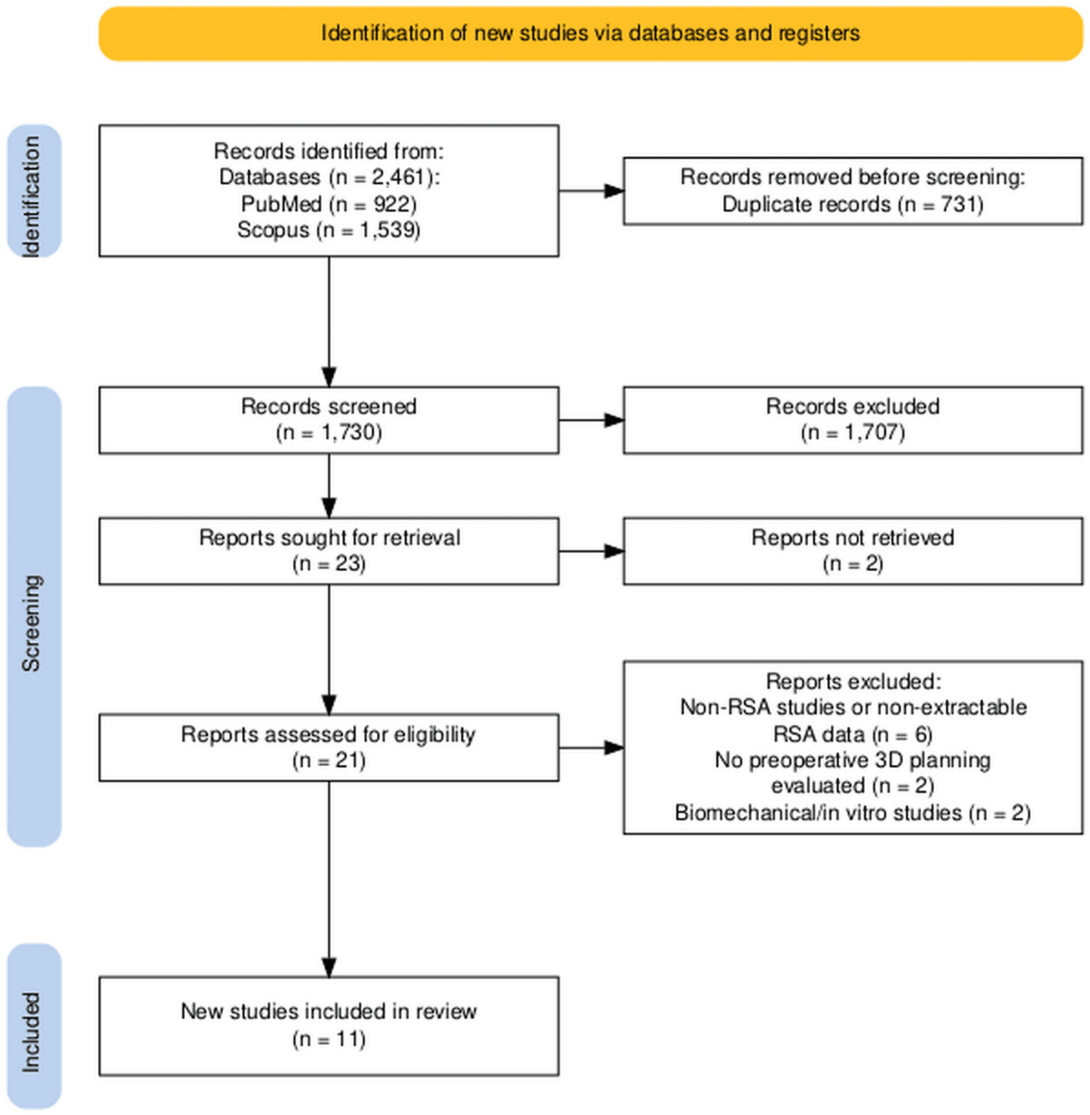
PRISMA 2020 flow diagram for the search strategy used to review three-dimensional planning in reverse shoulder arthroplasty PRISMA, Preferred Reporting Items for Systematic Reviews and Meta-Analyses; RSA, reverse shoulder arthroplasty.

The search initially attributed 2,461 studies. All duplicate records (n = 731) were identified and removed using the Mendeley Reference Manager programme. A total of 1,707 articles were excluded based on their title and abstract, leaving 23 articles. Of those, 11 studies were compatible with the eligibility criteria [10,12,17-25]. Figure 1 illustrates the PRISMA flow diagram.

Study Characteristics

All the 11 included studies were published between 2021 and 2026 and involved data about 635 shoulders undergoing RSA. The studies were derived from six countries, namely, South Korea (n=4), the United States (n=2), Italy (n=2), France (n=1), Australia (n=1) and a multicentre study from Switzerland (n=1). Regarding study design, two randomized controlled trials, two prospective non-randomized trials, and seven retrospective studies of various designs (comparative studies, cohort studies, case-control studies, and case series) were included. The sample size ranged from 15 to 135 shoulders per study. Follow-up duration was inconsistently reported across the included studies and ranged from 24 to 62 months, with only three studies providing these data [10,19,23]. The mean follow-up period was 42.6 months. Overall, the included studies involved patients undergoing RSA for various indications. The most common indication was rotator cuff tear arthropathy, which was reported in most studies [10,17-23]. Other indications included primary glenohumeral osteoarthritis [18,19,24], severe glenoid deformities or bone loss [12,17,25], proximal humeral fractures [18,21,24], and aseptic necrosis of the humeral head [21].

The 3D-evaluated preoperative planning technologies showed significant heterogeneity among studies. Seven studies investigated the use of PSI for glenoid base and/or screw placement [10,12,18-21,24]. Two studies evaluated real-time navigation or guidance systems, such as augmented reality technologies [22,23]. Two studies focused exclusively on 3D preoperative planning without the use of PSI or navigation systems [17,25]. Finally, one study incorporated augmented reality guidance [22]. Table 1summarizes the characteristics of all the studies included.

**Table 1:** Characteristics of the included studies RSA, reverse shoulder arthroplasty; BIO-RSA, bony increased-offset reverse shoulder arthroplasty; PSI, patient-specific instrumentation; AR, augmented reality; CT, computed tomography; OA, osteoarthritis; AVN, avascular necrosis; ROM, range of motion; ER, external rotation; PROMs, patient-reported outcome measures; OSS, Oxford Shoulder Score; ASES, American Shoulder and Elbow Surgeons Score; CMS, Constant-Murley Score; DASH, Disabilities of the Arm, Shoulder and Hand score; RCT, randomized controlled trial; IDI, image-derived instrumentation.

Study	Country	Study Design	Sample/Indication	Intervention	Comparator	Outcomes Measured	Key Findings
Tashjian et al., 2021 [17]	USA	Retrospective case series	N=15; mean age 75 years; RSA with structural glenoid bone grafting; severe glenoid erosion (Walch B2/B3, Favard E1–E3)	3D preoperative planning (Glenosys/Imascap software) without PSI; printed 2D plan used intraoperatively; Aequalis Reversed (Wright Medical) with BIO-RSA or structural graft	None (single-arm); planned vs. achieved corrections	Planned vs. achieved retroversion and inclination correction; planned vs. achieved graft thickness	Retroversion was significantly undercorrected (planned 18°±11° vs. achieved 12°±11°, P<0.001). Inclination correction was accurate (P=0.803). Failures (>10° deviation) occurred exclusively in severe B3/E3 deformities, suggesting PSI may be warranted in these cases.
Kwak et al., 2022 [18]	South Korea	Retrospective comparative study	N=39 shoulders (PSI n=19; conventional n=20); mean age 74–77 years; cuff tear arthropathy, OA, fracture	3D-printed PSI guides for central guide pin and peripheral screw placement in RSA; preoperative CT-based 3D planning	Conventional free-hand RSA with standard instrumentation	Baseplate version, inclination, translation, and rotation; screw length and angle accuracy; suprascapular/spinoglenoid notch involvement	PSI significantly improved accuracy of screw and baseplate positioning, particularly rotation (4.5° vs. 10.6°, P<0.001) and inclination (1.7° vs. 3.7°, P=0.018). Spinoglenoid notch involvement was lower with PSI (2 vs. 10 cases, P=0.014), suggesting reduced neurovascular injury risk.
Boekel et al., 2023 [19]	Australia	Prospective single-blinded RCT	N=47 (IDI n=24, conventional n=23); mean age ~69.5 years; primary OA and cuff arthropathy; 2-year follow-up	IDI/PSI: 3D CT-derived custom guide (Materialise) for guidewire placement; Lima SMR RSA implant	Conventional instrumentation with 3D preoperative planning (no PSI guide); same implant system	Guidewire/baseplate version and inclination accuracy; translational accuracy (≤2 mm threshold); clinical PROMs (OSS, ASES, CMS); scapular notching	IDI significantly improved guidewire placement in the superior/inferior plane (22/24 vs. 14/23 within 2 mm, P=0.01). IDI showed lower error with retroversion ≥10° (P=0.047). No significant differences in overall inclination or version. OSS at 2 years was significantly better with IDI (P=0.04). Scapular notching rates were comparable.
Gauci et al., 2024 [10]	France	Retrospective comparative study	N=37 (Inlay-155° n=21, Onlay-145° n=16); mean age 76±7 years; cuff tear arthropathy; BIO-RSA with PSI; mean follow-up 66±22 months	3D preoperative planning (Blueprint/Imascap) with 3D-printed PSI glenoid guide; ROM simulation; Aequalis Reverse II (Tornier) with BIO-RSA graft	Head-to-head: Inlay-155° vs. Onlay-145° humeral stems; simulated vs. actual clinical ROM	Active forward elevation, abduction, ER (planned vs. actual); radiological: RSA angle and lateralization shoulder angle	Simulated ROM differed significantly from clinical ROM (P<0.001): forward elevation and abduction were underestimated, ER overestimated. However, simulated differences between Inlay and Onlay designs were clinically confirmed. PSI achieved accurate glenoid positioning (no significant planned vs. postoperative difference).
Yelton et al., 2024 [12]	USA	Retrospective case-control study	N=45 (PSI n=22, non-PSI n=23); mean age ~72 years; primary RSA with severe glenoid deformity requiring bone grafting (BIO-RSA)	3D preoperative planning with PSI (Blueprint, Stryker); Stryker PSI guide for central pin placement; Aequalis Reversed (Wright Medical) with bone grafting	3D preoperative planning with standard instrumentation (no PSI guide); printed 2D plan used intraoperatively	Deviation from planned inclination and version (postoperative 2D CT); outlier rates (>10° error)	No significant difference between PSI and non-PSI in inclination (5.49°±3.72° vs. 6.91°±5.05°, P=0.437) or version error (8.37°±5.70° vs. 5.37°±4.43°, P=0.054). Greater planned version correction correlated with greater error specifically in the PSI group, indicating PSI may be less reliable for large corrections in severe deformity.
Kim et al., 2025 [20]	South Korea	Prospective multicentre single-blinded RCT	N=106 (PSI n=53, free-hand n=53); mean age ~75 years; cuff tear arthropathy, irreparable cuff tears, OA with cuff tear, severe glenoid deformity	3D CT-based preoperative planning with 3D-printed PSI guides (Anymedi software) for central pin and peripheral screw placement; Exactech RSA system	Conventional free-hand RSA with 3D preoperative planning only (no PSI)	Primary: proportion with deviation error ≥5° (version/inclination) or ≥2.5 mm (offset). Secondary: continuous deviations; peripheral screw accuracy; subgroup by glenoid morphology	PSI did not improve baseplate positioning (version 5°±5° vs. 5°±5°, P=0.901; inclination 6°±5° vs. 5°±4°, P=0.309). However, PSI significantly improved peripheral screw accuracy for anterior (P=0.041), superior (P=0.008), and inferior screws (P<0.001). In B2/B3 glenoids, inclination error was paradoxically larger with PSI.
Lee W et al., 2025 [21]	South Korea	Retrospective case series	N=30; mean age 73.5±8.5 years; 5 men/25 women; irreparable cuff tears, cuff tear arthropathy, OA, fracture, AVN	3D-printed PSI for baseplate and screw placement in RSA; CT segmentation (Mimics/3-Matic, Materialise); SLA-printed guides (Sindoh A1+); Equinoxe Reverse (Exactech)	None (single-arm); planned vs. actual implantation assessed	Baseplate version, inclination, and rotation errors; guide pin translation; screw angulation and length errors; screw safety (notch involvement)	High reproducibility: mean version error 2.7°±5.8° (83.3% within 10°), inclination error 0.9°±3.5° (100% within 10°). Guide pin translation was 1.7±1.0 mm (70% within 2 mm). The posterior screw was abandoned in 93.3% of cases due to insufficient bone or nerve proximity. No PSI-related complications.
Rojas et al., 2025 [22]	Multicentre (Switzerland, UK, Chile, Australia)	Prospective multicentre proof-of-concept case series	N=17; mean age 72.8±9.1 years; cuff tear arthropathy (65%), OA (29%), inflammatory arthropathy (6%)	3D preoperative planning (MyShoulder, Medacta) with intraoperative AR navigation (NextAR, Medacta) via head-mounted display; coracoid-mounted tracker; Medacta RSA system	None (single-arm); planned vs. intraoperative vs. postoperative CT values compared	Baseplate inclination, version, entry point, and reaming depth deviations (planned-intraoperative-postoperative); tracker/registration time; surgical time	AR navigation yielded low deviations: planned-to-postoperative mean 2.5°±3.2° inclination, 3.4°±4.6° version, 2.0±2.5 mm entry point. Outlier rate was 17.6% (vs. 48% reported for standard guides). No intraoperative complications. The mean time required for tracker placement and the registration process was 11 extra minutes. Total surgical time was less than 150 minutes in all cases.
Troiano et al., 2025 [23]	Italy	Retrospective comparative study	N=80 (navigation n=53, conventional n=27); mean age 74±5.7 years; eccentric OA or cuff tear arthropathy; mean follow-up 41.9±23.6 months	3D preoperative planning with intraoperative navigation (ExactechGPS, BlueOrtho); scapular tracker on coracoid; real-time guidance; Equinoxe Reverse (Exactech)	Conventional RSA with 3D preoperative planning (no navigation); same implant system	Clinical: CMS, DASH. Radiological: scapular notching, loosening. Surgical: operative time, screw number and length, complications	No significant differences in clinical outcomes (CMS: 66±16 vs. 68±16, P=0.56; DASH: 18±18 vs. 26±23, P=0.28). Navigation group used fewer screws (2±0.5 vs. 2.6±0.8, P=0.005) of greater length (36±5 vs. 31±4 mm, P<0.0001), potentially preserving bone stock. Complication rates were comparable. The navigationgroup exhibited an average increase of 7 min per procedure.
Yoon et al., 2025 [24]	South Korea	Retrospective multicentre comparative study	N=135 (PSI n=65, conventional n=70); mean age PSI 72.9±5.7 years, conventional 76.0±7.4 years; irreparable cuff tears, OA, fracture	3D-printed PSI guides (SEEANN Reconeasy software) for central pin and peripheral screw placement in RSA; biocompatible material (MED-AMB 10); four implant systems used	Conventional free-hand RSA with standard screw guides; same implant systems and surgeons	Baseplate version, inclination, and entry point deviations; peripheral screw angle and length deviations; screw prominence; screw safety outcomes	PSI significantly improved baseplate positioning: version 1.5°±1.1° vs. 6.7°±5.4° (P<0.001); inclination 2.8°±1.5° vs. 5.3°±3.7° (P=0.012); entry point 1.9±1.2 mm vs. 3.2±1.5 mm (P=0.037). PSI improved screw accuracy and had fewer screw prominences (9 vs. 23) and no insertion failures (vs. 7).
Donà et al., 2026 [25]	Italy	Retrospective single-centre observational study	N=84 RSA cases; primary glenohumeral OA (57.1%), cuff tear arthropathy (28.6%), massive cuff tear (14.3%); mean age 71.5±6 years	3D CT-based preoperative planning using BluePrint software; short uncemented 135° NSA stem (Perform, Wright Medical); glenoid and humeral component templating	Planned implant sizes vs. intraoperatively implanted components (no external control group)	Concordance of planned vs. implanted humeral stem, glenoid baseplate and glenosphere size; Cohen’s kappa agreement; stem alignment; complications	Full concordance between planned and implanted components in 23.1% of cases. Baseplate prediction accuracy was high (92.6%), glenosphere concordance moderate (60.7%), while humeral stem prediction accuracy was low (28.6%). A statistically significant correlation was observed between changes in humeral stem and glenosphere size (p < 0.05). No complications reported.

Risk of Bias Assessment

The two randomized controlled trials were assessed using the RoB 2 tool and were classified overall as having low risk of bias in all areas examined. The remaining nine non-randomized studies were assessed using NOS, receiving an overall score of 6 to 8 stars. Four studies received 6 stars, four studies 7 stars, and one study 8 stars, indicating overall moderate to good methodological quality. The lower score was mainly attributed to the absence of comparison groups in several studies, while most studies demonstrated adequate participant selection and complete outcome assessment. The risk of bias assessment, using RoB 2 and NOS, is presented in Tables 2, 3, respectively.

**Table 2:** Risk of bias assessment using the Cochrane RoB 2 (Risk of Bias 2) for the included studies

Study	Randomization Process	Deviations From Intended Interventions	Missing Outcome Data	Measurement of Outcomes	Selection of the Reported Result	Overall Risk of Bias
Boekel et al., 2023 [19]	Low risk	Low risk	Low risk	Low risk	Low risk	Low risk
Kim et al., 2025 [20]	Low risk	Low risk	Low risk	Low risk	Low risk	Low risk

**Table 3:** Risk of bias assessment using the Newcastle-Ottawa Scale (NOS) for the included studies

Study	Selection	Comparability	Outcome	Total
Tashjian et al., 2021 [17]	3	0	3	6
Kwak et al., 2022 [18]	3	1	3	7
Gauci et al., 2024 [10]	3	1	3	7
Yelton et al., 2024 [12]	3	1	3	7
Lee et al., 2025 [21]	3	0	3	6
Rojas et al., 2025 [22]	3	0	3	6
Troiano et al., 2025 [23]	3	1	3	7
Yoon et al., 2025 [24]	4	1	3	8
Donà et al., 2026 [25]	3	0	3	6

Radiological Outcomes

The accuracy of glenoid component placement was the most frequently reported radiological outcome and was evaluated in nine studies [12,17-22,24,25]. Radiological outcomes mainly included discrepancies in glenoid base version, inclination, entry point, and screw placement positioning between comparison groups or agreement between preoperative planning and final implant positioning.

Six comparative studies evaluated the effect of PSI or an intraoperative navigation system on the accuracy of glenoid component placement [12,18-20,24,25]. Of these, four studies reported a statistically significant improvement in at least one key placement parameter [18,19,24,25], while two studies did not demonstrate a significant superiority of PSI in terms of the primary assessment of glenoid base position [12,20]. However, even in the latter studies, several improvements were reported. The largest difference in favour of PSI was observed in the study by Yoon et al., where the version error decreased from 6.7°±5.4° to 1.5°±1.1° and the inclination error from 5.3°±3.7° to 2.8°±1.5° [24]. Similarly, Kwak et al. reported smaller version (1.7° vs. 3.7°) and inclination (1.7° vs. 3.7°) errors, while Boekel et al. demonstrated a significantly higher rate of placements within pre-specified deviation limits using image-derived instrumentation [18,19].

The three non-comparative studies that evaluated exclusively 3D planning, PSI, or navigation/augmented reality systems reported high agreement between preoperative planning and final glenoid component position [17,21,22]. In these studies, mean deviations in glenoid version ranged from 2.7° to 6° and mean deviations in glenoid inclination ranged from 0.9° to 2.5°. The frequency of significant deviations remained low, supporting the ability of 3D planning technologies to achieve reliable reproduction of the preoperative plan. The exact agreement between preoperative planning and final implantation, expressed as a percentage, was evaluated only by Donà et al. [25]. In this study, the prediction accuracy was high for the baseplate (92.6%) and moderate for the glenosphere (60.7%), while the agreement for the humeral stem was significantly lower (28.6%). The findings suggest that 3D planning appears more reliable for planning the glenoid facet compared to humeral implants.

The assessment of glenoid entry point accuracy was explicitly performed in three studies [19,22,24]. Rojas et al. found a mean deviation of 2.0±2.5 mm between the planned and postoperative guidewire entry point [22]. Similarly, the comparative studies of Boekel et al. and Yoon et al. advocated statistically significant improvement in entry point placement, when 3D preoperative planning and PSI were combined, in comparison with their control groups.

The accuracy of peripheral screw placement was assessed in five studies [18,20,21,23,24]. Generally, the available data suggest that the use of 3D preoperative planning technologies and customized guides contributes to improving the accuracy of screw placement and may allow the use of longer screws without increasing neurovascular compromise.

In comparative studies, Kwak et al. and Yoon et al. reported improved accuracy of peripheral screw placement with the use of PSI, while Kim et al. found significantly smaller placement errors for anterior, inferior, and superior screws [18,20,24]. In addition, Troiano et al. showed that the use of navigation systems was associated with a longer mean screw length compared with conventional planning (36±5 vs. 31±4 mm, P<0.001) [23]. Non-comparative studies confirmed the high reproducibility of preoperative planning in terms of screw trajectory, with low deviations and limited technical problems during implantation. Overall, the findings demonstrate a consistent trend in favour of 3D planning technologies for improving the placement and orientation of peripheral screws.

The available radiological data suggests that 3D preoperative planning technologies, particularly when combined with PSI or image-derived instrumentation, tend to improve the accuracy of glenoid component placement compared with conventional techniques, although the extent of benefit was not uniform across all studies.

Functional Outcomes

Functional outcomes were evaluated in only three studies [10,19,23], using heterogeneous outcome measures, including various patient-reported outcome measures (PROMs) and range of motion (ROM).

Boekel et al. reported significant improvements in all functional scores after surgery in both groups. When comparing image-derived instrumentation with conventional technique, no significant differences were observed in Constant-Murley Scores (CMS) and American Shoulder and Elbow Scores (ASES), while a statistically significantly higher Oxford Shoulder Score (OSS) was found in the image-derived instrumentation group at two years (43.9 vs. 39.9, P=0.04). However, this difference did not exceed the minimal clinically significant threshold and was considered of limited clinical significance [19]. Gauci et al. evaluated the correlation between preoperatively simulated and actual postoperative ROM. Although postoperative ROM differed significantly from simulated values, significant correlations were observed for anterior elevation, abduction, and external rotation. The authors concluded that preoperative 3D planning can provide clinically useful predictions of postoperative ROM and support implant selection [10]. Troiano et al. compared navigated and non-navigated RSA and found no significant differences in either CMS (86±16 vs. 86±16, P=0.56) or the Disabilities of Arm, Shoulder, and Hand (DASH) score (18±18 vs. 26±23, P=0.28) [23]. Overall, the available data did not support consistent clinical superiority of different 3D planning technologies, including PSI, image-derived instrumentation, or navigation systems over traditional functional scores.

Operative Parameters and Complications

Surgical parameters were evaluated in a limited number of studies. Rojas et al. investigated the use of an augmented reality navigation system and reported a total surgical time below 150 minutes in all cases. However, the mean time required for tracker placement and the registration process was about 11 minutes, which is additional time to surgery without intraoperative navigation [22]. Troiano et al. found, respectively, a mean difference of seven minutes in surgical time between the navigation group and the conventional group, which may be due to extra registration time. However, this increase in operative time was not considered statistically or clinically significant [23].

Complications and outcomes related to potential reoperations were reported heterogeneously across studies. Kwak et al. reported a lower incidence of spinoglenoid notch involvement in the PSI group compared with the conventional technique [18]. Similarly, Yoon et al. reported fewer protruding screws and no placement failures in the PSI group, in contrast to the control group, where multiple insertion failures were observed [24]. Boekel et al. found no differences in the occurrence of scapular notching between image-derived instrumentation and the conventional technique at the two-year follow-up [19]. The study by Lee et al. reported cases of screw perforation of the posterior cortex, which were mainly attributed to limited available bone stock or proximity to neural structures, but no complications directly related to the use of PSI were recorded [21]. In addition, neither Rojas et al. nor Doña et al. reported major intraoperative or postoperative complications at follow-up [22,25]. Finally, Troiano et al. reported comparable complication rates between navigation and conventional techniques [23]. Conclusively, the available data suggest that 3D preoperative planning and guidance technologies may help reduce some complications associated with screw and implant placement. However, reporting of complications and reoperations was limited in most studies, making it difficult to draw firm conclusions about their long-term clinical impact.

Discussion

This study synthesized and assessed the available data on the application of 3D preoperative planning technologies in RSA. The most significant finding was that the use of these technologies was associated with improved placement accuracy of RSA implants, especially the glenoid component and peripheral screws. However, the existing literature on functional outcomes was less homogeneous and did not consistently support clinical superiority over conventional techniques. Furthermore, data on surgical parameters, such as complication and revision rates, were generally scarce, with some studies indicating potential surgical benefits accompanied by small increases in procedure duration.

Accurate placement of the glenoid component is one of the most important technical aspects of RSA, as suboptimal placement has been linked with an increased risk of scapular notching, aseptic loosening, worse functional outcomes, and decreased implant survival [26,27]. In about 90% of RSA cases requiring revision, the glenoid component is mispositioned [28]. Considering the importance of proper glenoid positioning, the increasing use of 3D imaging and specialized planning software aims primarily to achieve optimal placement of the glenoid component in patients with complex anatomy or significant bone loss [29,30]. The findings of the present systematic review support this approach, as the majority of studies demonstrated smaller deviations in the glenoid component version, inclination, and/or entry point of guidewires compared to the planned positioning.

Our findings are aligned with those of previous systematic reviews that have also investigated the role of 3D preoperative planning for RSA. Lilley et al., in their recent systematic review, highlighted the potential of 3D planning and patient-specific guides to achieve high concordance in implant positioning, but the limited number of comparative studies makes interpretation of their findings more cautious [31]. The systematic review and meta-analysis of Villatte et al. assessed the use of PSI and included a mixed population of both anatomic TSA and RSA. This study concluded that these technologies improve implantation accuracy, although this conclusion cannot be drawn with safety due to the lack of studies assessing long-term outcomes [32]. More recently, Lee et al. performed a systematic review and meta-analysis comparing PSI, navigation, and mixed reality technologies in radiological outcomes in terms of the glenoid component. The authors advocated that these methods significantly improved implantation accuracy compared to conventional techniques, also confirming the results of the present study [33]. However, unlike previous reviews that focused primarily on radiological parameters, the present study included only clinical studies and attempted a broader synthesis of available data, including functional outcomes, surgical parameters, complications, and the need for revisions.

Despite the relatively consistent improvement in accuracy of implant placement, the effect of these 3D technologies on clinical and functional outcomes remains unclear. Most individual studies assessing 3D technologies as planning tools focus on radiological data, and this may be attributed to the early stages of these modalities [34]. In our systematic review, only three of 11 studies report such data [10,19,23]. Interestingly, these studies showed CMS, ASES, and DASH scores comparable to those of standard techniques [19,23]. Boekel et al. advocated statistically significant differences in OSS in favour of 3D planning; however, this was not judged as clinically significant [19]. Gauci et al. indicated a useful ROM prediction but could not support a superior postoperative function [10]. One possible explanation why improved radiological outcomes with the use of 3D planning are not necessarily translated into superior clinical outcomes may be the multifactorial nature of RSA success. The final postoperative function can be influenced by several patient-related factors, including deltoid function, soft-tissue quality, preoperative ROM, prior medications, and rehabilitation protocols [35-37]. Moreover, most eligible studies had a relatively short follow-up period. For this reason, complications, such as aseptic glenoid loosening, humeral stem disassembly, or the need for revision, which require more time to manifest, may not be detected [38].

The findings of this systematic review should be interpreted with caution due to several limitations. The significant heterogeneity among the included studies regarding study design, preferred 3D technology, patient characteristics, and reported outcome measures did not allow for quantitative data analysis. In addition, the sample sizes and follow-up periods of the included studies were relatively short. Most of the eligible studies were retrospective and non-randomized, so selection bias is also possible. Finally, the inconsistency in reporting of complications, implant survival rates, and clinical functional outcomes makes the assessment of the effects of 3D planning extremely challenging.

## Conclusions

3D preoperative planning technologies, with or without PSI, navigation systems, or other guidance technologies, can improve the accuracy of implant positioning in RSA, particularly for the glenoid component and peripheral screws. These advantages were particularly evident in radiological outcomes, with most studies reporting small deviations from preoperative planning and improved implant placement in comparison with conventional practice. On the contrary, improved surgical accuracy was not consistently accompanied by superior clinical and functional outcomes, and available data on operative time, complications, revisions, and long-term implant survival remain limited. Future research is required, optimally with a prospective design, control groups, and longer follow-up periods, to clarify whether the technical advantages of these modalities translate into substantial and sustainable clinical benefits.
